# Transcriptome-Wide Identification and Analysis Reveals m^6^A Regulation of Porcine Intestinal Epithelial Cells Under TGEV Infection

**DOI:** 10.3390/vetsci13010010

**Published:** 2025-12-21

**Authors:** Ying Liu, Gang Zhou, Guolian Wang, Zhengchang Wu

**Affiliations:** 1 School of Life Science, Huaiyin Normal University, Huaian 223001, China; yingliu@hytc.edu.cn; 2 Huaiyin Institute of Agricultural Sciences in Xuhuai Regio, Huaian 223000, China; yddkly@163.com; 3 Key Laboratory for Animal Genetics, Breeding, Reproduction and Molecular Design of Jiangsu Province, College of Animal Science and Technology, Yangzhou University, Yangzhou 225009, China

**Keywords:** transmissible gastroenteritis virus, N^6^-methyladenine, IPEC-J2, MeRIP-seq

## Abstract

Transmissible gastroenteritis virus (TGEV) represents a critical intestinal pathogen responsible for acute enteritis in pigs, posing significant challenges to global swine production biosecurity. N^6^-methyladenosine (m^6^A), the most abundant epitranscriptomic mark in eukaryotic messenger RNA, has emerged as a regulatory factor in host–virus interactions. Despite its recognized importance, the functional significance of m^6^A modifications during TGEV infection of porcine jejunal epithelial (IPEC-J2) cells remains unexplored. Here, we established a TGEV-infected IPEC-J2 cell model and we employed methylated RNA immunoprecipitation sequencing (MeRIP-seq) to comprehensively profile the m^6^A epitranscriptomic landscape and identify N^6^-methyladenosine-bearing transcripts in IPEC-J2 cells following TGEV challenge. A total of 14,813 m^6^A peaks were identified in the IPEC-J2, distributed in 7728 genes, mainly enriched in the CDS and 3′-UTRs. After TGEV infection, we identified 832 m^6^A peaks and 1660 genes with significant changes. Integrative analysis revealed a direct positive relationship between N^6^-methyladenosine modification abundance and transcript expression levels. Through integrated examination of MeRIP-Seq and RNA-Seq datasets, we identified 105 transcripts bearing m^6^A modifications, which were mainly enriched in the mTOR signaling pathway. Protein–protein interaction (PPI) network and RT-qPCR analysis demonstrated that *SOS2* probably acts an important moderator in TGEV infection. This work contributes to understanding the m^6^A modification landscape in the TGEV-swine model and suggests *SOS2* as potential target for future antiviral strategies.

## Simple Summary

Transmissible gastroenteritis virus (TGEV) represents a widespread and critically pathogenic enteric pathogen that poses significant economic threats to pig production worldwide. However, the role of m^6^A modification in TGEV-infected host cells remains largely unexplored. In this study, we conducted a comprehensive analysis of m^6^A epitranscriptomic modifications in TGEV-challenged IPEC-J2 cell lines. The resulting data constitute the first systematic characterization of N^6^-methyladenosine landscapes in porcine intestinal cells experiencing pathogen-driven metabolic shifts, with implications for targeted therapeutic interventions against this economically significant virus.

## 1. Introduction

Transmissible gastroenteritis (TGE) represents an acute and highly infectious enteric disease affecting swine populations, with the transmissible gastroenteritis virus (TGEV) serving as its etiological agent, which remains a major threat to modern pig production systems [1]. Among porcine viruses, coronaviruses—particularly TGEV—have become valuable models for dissecting viral immune evasion strategies. TGEV shares common features in genome architecture and immune-modulatory pathways with other coronaviruses, including SARS-CoV-2, offering important clues to the conserved principles governing coronavirus immune escape [2]. At the same time, its strong enteropathogenicity and substantial impact on herd health and productivity underscore its importance, while the possibility of interspecies transmission further elevates its epidemiological relevance [3]. The structural proteins of TGEV not only orchestrate key steps in the viral lifecycle, such as attachment, entry, assembly, and release, but also actively participate in subverting host innate immune defenses, forming the molecular foundation of its immune evasion capacity [4]. Viral transmission occurs predominantly via fecal–oral and aerosol routes, with the pathogen exhibiting selective tropism for intestinal villous epithelial cells lining the porcine jejunum and ileum [5]. Viral replication leads to epithelial necrosis, villus blunting, and mucosal injury, which compromise intestinal integrity. As a result, infected piglets typically develop acute vomiting, severe yellow–green watery diarrhea, profound dehydration, and malabsorptive symptoms [6,7]. Newborn swine with immunologically immature systems bear the most severe disease burden, experiencing rapid clinical deterioration that culminates in fatality rates spanning 80–100% within the opening 10 days of life [8]. Compounding these challenges, ongoing viral evolution and genetic recombination continue to threaten vaccine effectiveness and reinforce concerns about cross-species transmission [4]. Consequently, clarifying the molecular events that govern TGEV replication and identifying new host factors that restrict infection are essential steps toward developing effective strategies for disease prevention and control.

RNA modifications exert critical influence over RNA stability, function, and metabolic fate. Among the diverse chemical marks identified to date, five major forms of RNA methylation have been characterized: 5-methylcytosine (m^5^C), N^7^-methylguanine (m^7^G), N^6^-methyladenosine (m^6^A), N^1^-methyladenosine (m^1^A), and pseudouridine (ψ) [9]. Of these, m^6^A is the most abundant and biologically significant internal RNA modification in eukaryotes, acting as a central regulator of post-transcriptional gene expression [10]. Typically, m^6^A residues are enriched near stop codons, transcription start sites (TSSs), coding sequences (CDSs), and both 5′- and 3′-untranslated regions (UTRs) of modified transcripts [11]. Through these localized deposits, m^6^A profoundly influences RNA splicing, nuclear export, translation efficiency, and decay dynamics [12]. Consequently, m^6^A is indispensable for a range of physiological events—such as gametogenesis and cell proliferation—while also contributing to pathological states including tumorigenesis and infertility [13]. Emerging evidence further suggests that m^6^A participates in viral replication cycles [14] and plays integral roles in regulating host–pathogen interactions [15,16]. Although extensive research on m^6^A regulation has been conducted in humans and rodent models, substantially fewer studies have investigated its relevance in livestock species. In pigs, existing work has mainly examined the role of m^6^A methylation in adipogenesis [17], lipid metabolic processes [18], stem cell fate decisions [19], and liver development [20]. Like DNA and protein modifications, m^6^A marks are dynamically reversible post-transcriptional regulators that modulate mRNA expression, structure, splicing patterns, stability, half-life, and translational outcomes [21,22]. Increasing evidence also implicates m^6^A in bacterial and viral pathogenesis, gastrointestinal disorders, and the orchestration of host immune responses [23,24]. Despite these insights, comprehensive transcriptome-level profiling of m^6^A during porcine infectious diseases remains scarce, highlighting an important gap in epigenetic research on livestock of agricultural significance. Previous investigations into TGEV infection in porcine intestinal epithelial cells (IPEC-J2) have identified gene- and metabolite-level responses using integrated metabolomic and transcriptomic approaches [25,26]. In addition, genome-wide DNA methylation patterns have been documented in porcine testicular cells following TGEV challenge [27]. Nevertheless, the global m^6^A landscape in TGEV-infected IPEC-J2 cells has not yet been explored. To address this gap, We employed methylated RNA immunoprecipitation in conjunction with next-generation sequencing (MeRIP-seq) [28] to define transcriptome-wide m^6^A methylation profiles in control and TGEV-treated cells. This analysis revealed distinct m^6^A modification signatures associated with TGEV infection. Subsequent bioinformatic analyses and qRT-PCR validation further uncovered key genes and signaling pathways responsive to TGEV exposure in IPEC-J2 cells. Collectively, these findings provide an integrated m^6^A-epitranscriptomic, transcriptomic, and metabolomic framework that deepens our understanding of host cellular responses and sheds new light on the molecular basis of TGEV pathogenesis.

## 2. Materials and Methods

### 2.1. Cell Culture and TGEV Infection

IPEC-J2 cells, obtained from the University of Pennsylvania (Philadelphia, PA, USA), were cultured in DMEM/F12 medium containing 10% fetal bovine serum under standard conditions (37 °C, 5% CO_2_, humidified environment). To establish the infection model, cells were inoculated into 96-well plates at 2 × 10^4^ cells/well, permitted to attach for 12 h, then challenged with TGEV (SHXB strain) for durations of 24, 48, or 72 h.

For m^6^A-seq analysis, IPEC-J2 cells were cultured until reaching ~70% confluence, then exposed to TGEV at MOI = 1 (infected cohort) or treated with an equal volume of DMEM as a negative control (mock cohort). Following a 1 h viral attachment phase at 37°C, the inoculum was aspirated, and monolayers underwent three sequential washes with phosphate-buffered saline (PBS; Biosharp, Beijing, China). Cells were then maintained in DMEM supplemented with 2% FBS for a further 48 h at 37 °C before harvesting for RNA isolation.

### 2.2. TCID_50_ Assay

IPEC-J2 cells were seeded into 96-well microplates and propagated to ~90% confluency. Serial ten-fold virus dilutions were subsequently generated and inoculated onto the monolayers, with a 1 h attachment phase at 37 °C. Following aspiration of the viral inoculum, fresh medium was supplied, and cultures were monitored at 24, 48, and 72 h post-infection. Cytopathic effects (CPE) were documented at daily intervals, and the 50% tissue culture infectious dose (TCID_50_) was calculated via the Reed-Muench statistical method based on CPE-positive well frequencies.

### 2.3. m^6^A MeRIP-Seq and RNA-Seq

For transcriptomic profiling, triplicate biological samples were harvested from both mock-treated and TGEV-challenged cell populations. RNA isolation was accomplished using TRIzol reagent (Invitrogen, Carlsbad, CA, USA), with subsequent assessment of RNA quality and yield via NanoDrop ND-1000 spectrophotometry (Thermo Fisher Scientific, Waltham, MA, USA). CloudSeq Biotech Inc. (Shanghai, China) executed MeRIP-seq and RNA-seq analyses adhering to established methodologies [28]. Specifically, N6-methyladenosine immunoenrichment was performed employing the GenSeq™ m^6^A-MeRIP Kit (GenSeq Inc., Dublin, China) per manufacturer specifications. Sequencing libraries were constructed from both immunoprecipitated (IP) fractions and matched input RNA using the NEBNext^®^ Ultra II Directional RNA Library Prep Kit (New England Biolabs, Ipswich, MA, USA). Library quality control and fragment size profiling were conducted on an Agilent 2100 Bioanalyzer (Agilent Technologies, Santa Clara, CA, USA), followed by sequencing on the Illumina HiSeq 4000 platform (Illumina, San Diego, CA, USA) generating 150 bp paired-end reads. Complete raw sequencing data for m^6^A-seq and RNA-seq have been archived in the NCBI Sequence Read Archive under BioProject accession PRJCA051364.

### 2.4. Sequencing Data Analysis

Following sequencing, Q30 quality metrics were applied to assess data reliability. Adapter sequences at the 3′ end and low-quality reads were removed using Cutadapt v1.9.3 [29]. The resulting clean reads were then aligned to the pig reference genome (*Sscrofa11.1*; https://www.ncbi.nlm.nih.gov/genome/?term=pig (accessed on 18 December 2025)) using Hisat2 v2.0.4 [30]. m^6^A-enriched peaks in the MeRIP-seq datasets were identified with MACS2 v2.1.2 [31], after which peaks overlapping annotated transcript exons were retained for downstream analyses. Differential methylation between control and TGEV-infected samples was evaluated using diffReps [32], with significant peaks defined by |log_2_ fold change| ≥ 1 and FDR ≤ 0.05. These differentially methylated peaks were subsequently subjected to Gene Ontology (GO) and Kyoto Encyclopedia of Genes and Genomes (KEGG) enrichment analyses to determine their functional and pathway associations.

The distribution of m^6^A peaks across major transcript regions (TSSs, CDSs, stop codon (stopC), 5′- and 3′-UTRs) was examined, and peak localization along full-length transcripts was visualized using the Integrative Genomics Viewer (IGV) [33]. Enriched sequence motifs within the identified peaks were analyzed using the MEME Suite (http://meme-suite.org/, accessed on 12 August 2025). For transcriptome profiling, raw mRNA-seq reads were quantified using HTSeq (v0.9.1) and normalized with edgeR. Differentially expressed genes exhibiting altered m^6^A methylation were subsequently subjected to GO and KEGG pathway enrichment analyses to evaluate their functional and biological pathway associations.

### 2.5. qPCR Analysis

Total RNA isolation from IPEC-J2 cells was performed using TRIzol reagent (Invitrogen) following the protocol outlined above. First-strand cDNA synthesis was accomplished with the PrimeScript RT-PCR Kit (TaKaRa, Beijing, China). Real-time quantitative PCR amplification was executed on an ABI 7500 platform (Applied Biosystems, Waltham, MA, USA) employing the following thermal profile: initial denaturation at 95°C for 30 s, succeeded by 40 amplification cycles consisting of 95°C for 5 s and 60°C for 34 s. Expression levels of *SOS2*, *RAB7B*, *CHML*, *PAK6*, *LOC100152738*, *STX3*, *NIM1K*, *ARL9*, *RAB3IL1*, *TIAM1*, and *TGEV-N* were assessed, with *GAPDH* employed as the normalization reference. All primer sequences are detailed in Supplementary Table S1.

### 2.6. Western Blotting Analysis

Protein extraction was accomplished using the NE-PER Nuclear and Cytoplasmic Extraction Reagents kit (Thermo Fisher Scientific) according to supplier protocols. Protein quantification was performed via BCA assay (Nanjing Keygen Biotech, Nanjing, China). Following electrophoretic separation, equivalent protein quantities were electrotransferred to PVDF membranes and immunoblotted with primary antibodies targeting TGEV-N (Youlong Biotech, Shanghai, China) and GAPDH (Abbkine, Wuhan, China). Membranes were then exposed to horseradish peroxidase-conjugated goat anti-rabbit IgG secondary antibody (Abcam, Cambridge, UK; 1:5000 dilution), and protein signals were subsequently visualized.

### 2.7. Statistical Analyses

Relative gene expression was calculated employing the 2^−ΔΔCt^ method [34]. Statistical evaluation was conducted using SPSS software version 18.0 (SPSS, Inc., Chicago, IL, USA). Results are presented as mean ± standard deviation (SD), with group comparisons performed via Student’s *t*-test. Statistical significance was defined as *p* < 0.05.

## 3. Results

### 3.1. Establishment of TGEV-Infected IPEC-J2 Cell Model

**Figure 1:**
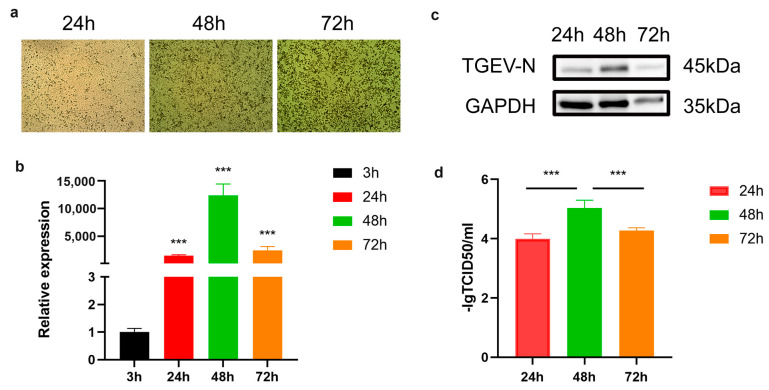
Establishment of TGEV-infected (MOI = 1) IPEC-J2 cell model. (**a**) Microscopic observation of IPEC-J2 cell morphology following TGEV infection across multiple time points (24, 48, and 72 h). (**b**) Relative TGEV-N transcript abundance in IPEC-J2 cells at indicated post-infection intervals. (**c**) Western blot analysis of TGEV-N protein expression in TGEV-challenged IPEC-J2 cells across different time points. (**d**) Viral titer determination via TCID_50_ assay. Data represent mean ± standard deviation (SD) from three independent experiments. *** *p* < 0.001.

To examine the replication dynamics and pathogenic effects of TGEV in host cells, an IPEC-J2 infection model was generated using TGEV and monitored at multiple time points (24, 48, and 72 h). With prolonged infection, IPEC-J2 cells exhibited progressive morphological deterioration, including cellular shrinkage, deformation, and eventual cell death (Figure 1a). qPCR and Western blot analyses were performed to evaluate TGEV-N expression over time. As shown in Figure 1b, TGEV-N mRNA levels rose steadily from 3 h to 48 h, reaching their maximum at 48 h and subsequently decreasing at 72 h. Consistently, Western blot data demonstrated an accumulation of TGEV-N protein up to 48 h (Figure 1c). TCID_50_ measurements further indicated a marked increase in viral titers at the 48 h time point (Figure 1d). Collectively, these findings confirm the successful establishment of the TGEV–IPEC-J2 infection model and identify 48 h post-infection as the optimal time point for subsequent analyses.

### 3.2. Transcriptome-Wide Characterization of m^6^A Modification Dynamics upon TGEV Infection

Transcriptome-wide m^6^A-seq and RNA-seq were conducted on TGEV-infected and control IPEC-J2 cells. The m^6^A-seq libraries produced 52.27–57.23 million clean reads, while 47.64–56.28 million clean reads were obtained from the RNA-seq libraries, which also served as the input controls for m^6^A profiling (Table S2). Among these clean reads, 95.18% and 95.25%, respectively, were successfully aligned to the Sscrofa11.1 reference genome, indicating high sequencing quality and reliable dataset integrity.

**Figure 2:**
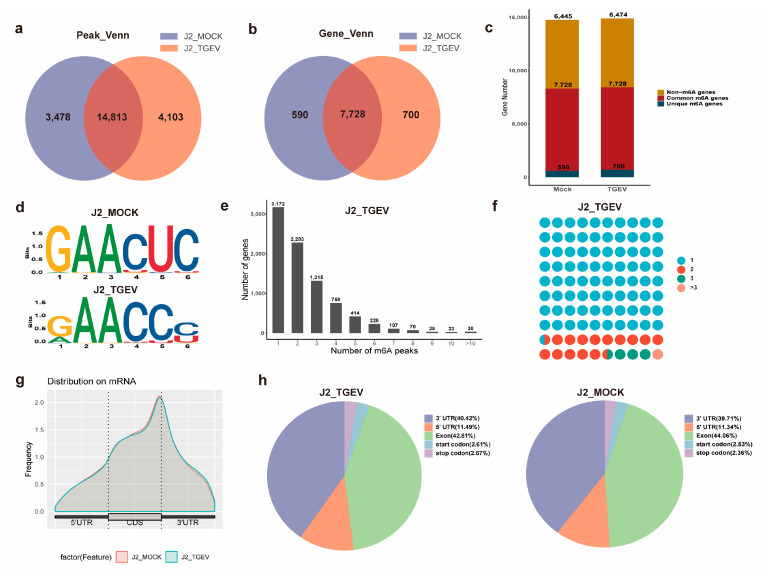
m^6^A-RIP-seq-mediated assessment of the m^6^A methylome of TGEV-treated IPEC-J2 cells. (**a**) Numbers of group-specific and common m^6^A peaks. (**b**) A Venn diagram was constructed to highlight m^6^A peaks present in TGEV-treated and control samples. (**c**) m^6^A-modified genes identified via m^6^A-seq. (**d**) A sequence diagram highlighting the conserved RRACH motif in m^6^A-containing peak regions. (**e**) Numbers of m^6^A-modified peaks per gene in TGEV-treated samples. (**f**) Distribution of m^6^A modification peaks across individual genes exclusively enriched in TGEV-infected samples. (**g**) Metagene profile depicting m^6^A peak density distribution. (**h**) Pie chart representation of m^6^A peak localization across distinct transcript regions in TGEV-infected versus mock-treated samples. J2_TGEV, TGEV-treated IPEC-J2 cells; J2_MOCK, untreated IPEC-J2 cells.

Model-based ChIP-seq (MACS) analysis identified 18,916 m^6^A peaks in TGEV-infected samples and 18,291 peaks in control samples (Figure 2a), corresponding to 8428 and 8318 m^6^A-modified genes, respectively (Figure 2b). Venn diagram analysis showed that 14,813 peaks and 7728 m^6^A-modified genes were shared between the two groups. Relative to the control cells, TGEV-infected cells displayed 3478 newly gained peaks and 4103 peaks that were absent after infection, indicating marked global alterations in m^6^A methylation patterns (Figure 2c). Furthermore, motif enrichment analysis revealed that m^6^A peaks in both groups predominantly contained the canonical GAACU/GAACC consensus sequence (Figure 2d).

To further characterize m^6^A deposition at the gene level, we analyzed the distribution of modification peaks across individual transcripts. Approximately 37.6% of m^6^A-modified genes (3172/8428) in the TGEV-infected group contained a single unique m^6^A peak. Overall, the majority of modified genes (5077/7970) harbored one to three m^6^A sites (Figure 2e). Notably, genes exhibiting TGEV-specific methylation changes predominantly contained only a single m^6^A peak, in contrast to the broader distribution observed among all m^6^A-modified genes (Figure 2f).

We further examined transcriptome-wide m^6^A peak distribution in both groups to identify potential treatment-specific patterns of methylation. Peaks were categorized according to their locations within transcripts, including the start codon (startC), 5′-UTR, coding sequence (CDS), stop codon (stopC), and 3′-UTR. Metagene profiling demonstrated that m^6^A signals were predominantly concentrated within the CDS and 3′-UTRs (Figure 2g). Both total and uniquely detected peaks showed marked enrichment around the CDS (exonic) and 3′-UTRs, highlighting these areas as major sites of m^6^A deposition (Figure 2h).

### 3.3. Pathway Enrichment Analysis of m^6^A-Methylated Genes

**Figure 3:**
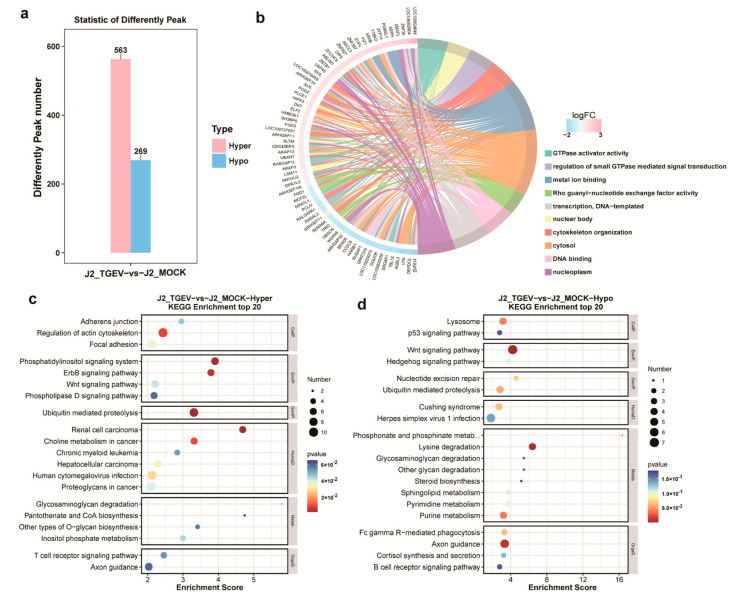
Global changes in m^6^A modification patterns in TGEV-treated IPEC-J2 cells relative to control cells. (**a**) Histogram highlighting distinct m^6^A peaks (fold change ≥ 2 and *p* < 0.05). (**b**) GO analysis of molecular_function, cellular_component, biological_process associated with transcripts exhibiting differential m^6^A peaks. (**c**,**d**) KEGG pathway analyses of transcripts with significantly up- or down-regulated m^6^A modification rates in TGEV-treated samples relative to control samples.

To evaluate infection-induced m^6^A dynamics, we examined peak abundance differences between TGEV-exposed and uninfected cells. Among 14,813 overlapping m^6^A modification sites, 832 exhibited significant differential methylation patterns. TGEV infection induced hypermethylation in 563 peaks and hypomethylation in 269 peaks (Figure 3a; Table S3). To elucidate the biological significance of these m^6^A alterations, GO and KEGG pathway analyses were performed on genes exhibiting differential methylation (DMGs). GO enrichment revealed that DMGs were predominantly associated with GTPase activator activity, metal ion binding, DNA binding, and Rho guanyl-nucleotide exchange factor activity under the Molecular Function category; nuclear body, cytosol, and nucleoplasm under Cellular Component; and regulation of small GTPase–mediated signal transduction, cytoskeleton organization, and DNA-templated transcription under Biological Process (Figure 3b). Further KEGG pathway analysis showed that genes containing hypermethylated m^6^A sites were enriched in pathways such as regulation of the actin cytoskeleton, phosphatidylinositol signaling, and ubiquitin-mediated proteolysis (Figure 3c). In contrast, transcripts with hypomethylated m^6^A sites were associated with pathways including the Wnt signaling pathway, axon guidance, and others (Figure 3d).

### 3.4. Identification of Transcriptional Changes in TGEV-Infected Cells

**Figure 4:**
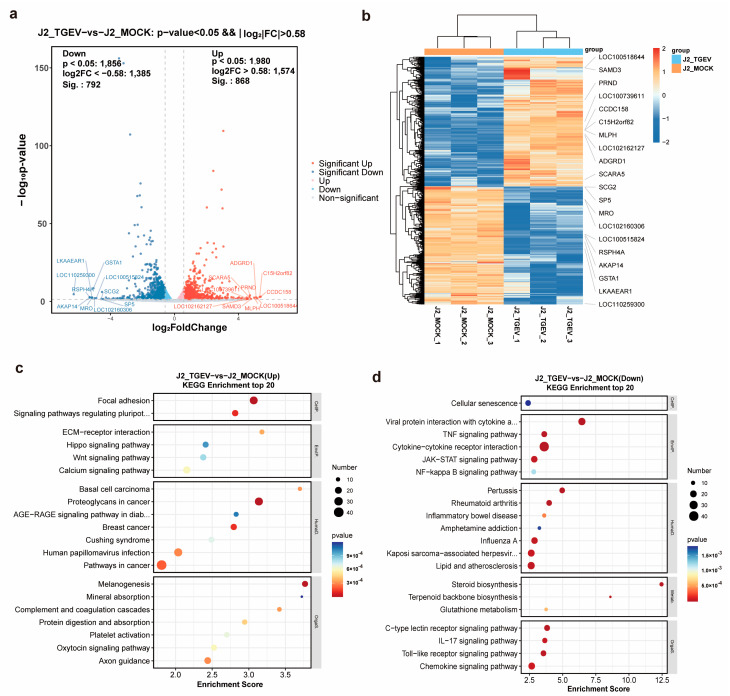
Identification of DEGs between TGEV-treated and control IPEC-J2 cells via RNA-seq. (**a**) Scatter plots showing DEGs in TGEV-treated and control cells. (**b**) Hierarchical clustering analysis of RNA-seq data from TGEV-treated and control cells. (**c**,**d**) KEGG pathway analysis of genes that were up- and downregulated in TGEV-treated samples relative to control samples.

The m^6^A-seq input libraries were also used as RNA-seq datasets to assess global transcriptional changes between TGEV-infected and control cells. Based on these RNA-seq profiles, scatter plot visualization and hierarchical clustering analyses were performed to compare gene expression patterns across the two groups (Figure 4a,b). A total of 1660 differentially expressed genes (DEGs) were detected, comprising 868 genes with elevated expression and 792 genes with reduced expression in TGEV-infected cells (Table S4). KEGG pathway enrichment revealed that upregulated transcripts were primarily enriched in calcium signaling and Wnt signaling pathways (Figure 4c), whereas the downregulated genes were mainly involved in cytokine–cytokine receptor interaction, TNF signaling, Toll-like receptor signaling, and other immune-related pathways (Figure 4d).

### 3.5. Conjoint Analysis of m^6^A-RIP-Seq and RNA-Seq Data

**Figure 5:**
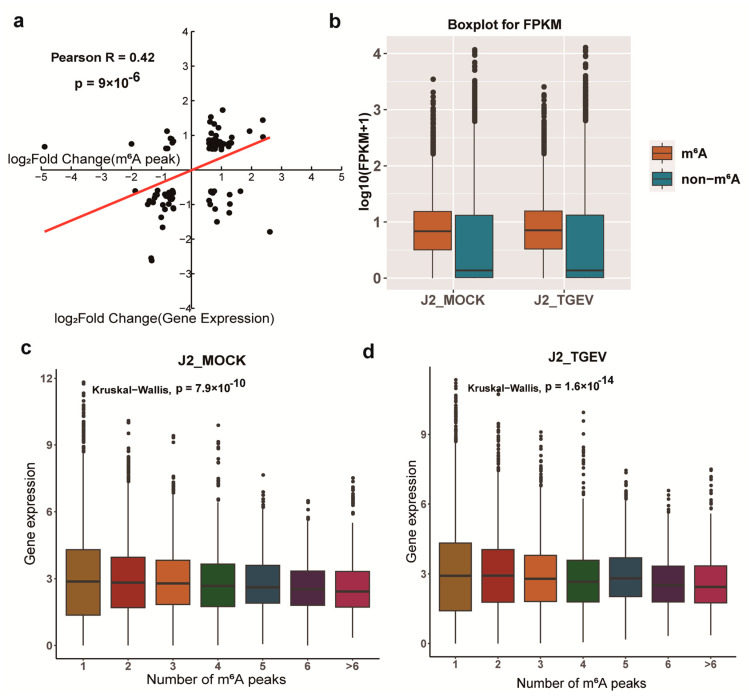
Association analysis between m^6^A-RIP-seq and RNA-seq in TGEV-treated cells. (**a**) Scatter plot analysis illustrating the positive relationship between m^6^A modification levels and corresponding transcript abundance. (**b**) Comparative expression profiles of m^6^A-bearing versus m^6^A-deficient transcripts across experimental conditions. (**c**,**d**) Relative mRNA expression levels for transcripts containing different numbers of m^6^A peaks.

To investigate the interplay between m^6^A modifications and transcript levels in TGEV-challenged cells, we performed integrative analysis of m^6^A-RIP-seq and RNA-seq data. This conjoint evaluation demonstrated a strong positive association between alterations in m^6^A methylation status and corresponding mRNA expression changes (R = 0.42, *p* < 0.0001; Figure 5a). We then examined the distribution of m^6^A peaks along transcripts and compared expression levels of m^6^A-modified versus non-modified genes in both TGEV-treated and control cells (Figure 5b). As noted earlier, individual genes contain varying numbers of m^6^A sites. Notably, genes harboring a greater number of m^6^A peaks tended to display higher expression levels, indicating an association between increased methylation density and transcriptional upregulation (Figure 5c,d).

**Figure 6:**
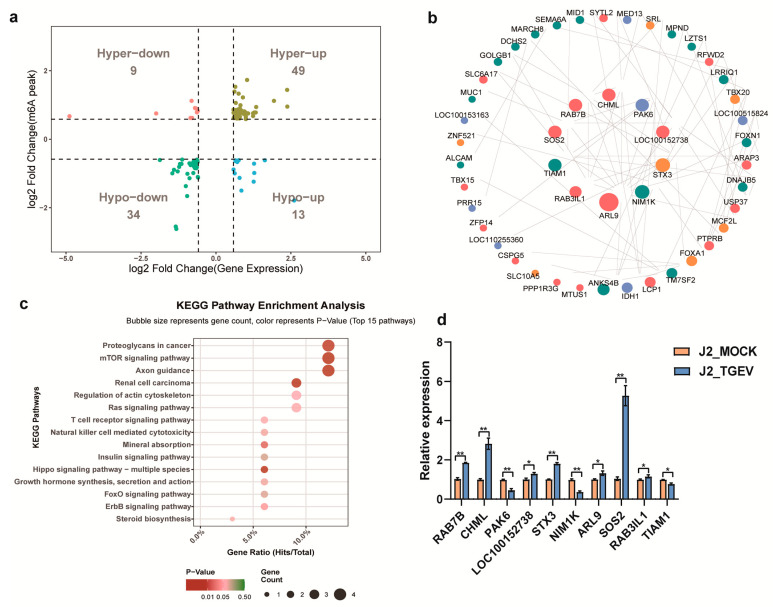
Conjoint analysis of m^6^A-RIP-seq and RNA-seq data. (**a**) A four-quadrant graph highlighting DEGs containing differentially methylated m^6^A peaks. (**b**) PPI network constructed with dysregulated m^6^A-modified genes. Cytoscape was used to visualize a network incorporating m^6^A-modified genes/proteins based on the conjoint analysis of m^6^A-RIP-seq and RNA-seq data. (**c**) KEGG pathway analysis of 105 m^6^A-modified genes. (**d**) qPCR validation of dysregulated m^6^A-modified genes. Relative mRNA expression levels of 10 representative genes were assessed using qPCR. Data represent mean ± standard deviation (SD) from three independent experiments. * *p* < 0.05, ** *p* < 0.01.

Combined analysis of m^6^A-RIP-seq and RNA-seq data demonstrated distinct associations between alterations in methylation status and corresponding mRNA expression levels (Figure 6a). Among the 563 hypermethylated m^6^A sites identified, 49 corresponding genes were upregulated (“hyper-up”), whereas 9 showed reduced expression (“hyper-down”). In contrast, among genes exhibiting hypomethylated m^6^A methylation, only 13 showed increased expression (“hypo-up”), whereas 34 displayed decreased expression (“hypo-down”) (Table S5). Notably, 49 of the 62 upregulated transcripts (79%) in TGEV-infected cells were associated with hypermethylated m^6^A peaks. Collectively, these findings indicate that increased m^6^A methylation is generally linked to enhanced gene expression during TGEV infection.

To further clarify the regulatory mechanisms underlying TGEV infection, we constructed a protein–protein interaction (PPI) network based on 105 m^6^A-modified genes (Table S5) by using the STRING database. As shown in Figure 6b, the resulting network consisted of 55 nodes and 79 interaction pairs and was visualized using Cytoscape 3.9.1 [35]. Among these nodes, SOS2, RAB7B, CHML, PAK6, LOC100152738, STX3, NIM1K, ARL9, RAB3IL1, and TIAM1 emerged as central hub proteins. Notably, SOS2 was identified as both a differentially expressed gene (DEG) and a differentially methylated gene (DMG) in the RNA-seq and m^6^A-seq datasets, respectively, and showed significant upregulation following TGEV infection (Table S4). KEGG pathway enrichment of the 105 candidate genes revealed several TGEV-associated pathways, with the mTOR signaling pathway standing out as the most relevant (Figure 6c; Table S6). To validate our sequencing findings, we examined the expression of several key network genes using qPCR, and the expression profiles were consistent with those obtained from RNA-seq analysis (Figure 6d). In particular, SOS2 expression was significantly elevated in TGEV-infected cells (*p* < 0.01). Collectively, these results indicate that *SOS2* probably serves as a crucial mediator in the pathogenesis of TGEV infection in IPEC-J2 cells.

## 4. Discussion

Post-transcriptional regulatory mechanisms exert pivotal control over RNA stability, maturation, and functionality. Viral RNA molecules are equally subject to such regulation, which profoundly influences their biological activities [36]. m^6^A modification on both viral and host transcripts has been shown to influence viral replication and host antiviral responses [37]. Williams et al. (2019) provided a comprehensive overview of this field, noting that m^6^A modification can enhance the replication of several RNA viruses (IAV, SV40, and RSV) although the underlying mechanisms differ among viruses [38]. Despite growing interest, the precise physiological and pathological consequences of m^6^A regulation during viral infection remain insufficiently understood in both in vitro and in vivo settings. Nevertheless, increasing evidence indicates that m^6^A significantly modulates the transcriptional behavior of numerous DNA and RNA viruses [39]. Because m^6^A methylation is an epigenetic mark that can be shaped by both viral and host machinery, elucidating its role in infection may offer new perspectives on viral evolution and host–pathogen interactions. However, the function of m^6^A methylation in regulating TGEV infection in IPEC-J2 cells has not been defined. In this study, we performed MeRIP-seq to map the m^6^A methylome in TGEV-infected IPEC-J2 cells. To the best of our knowledge, this study constitutes the inaugural genome-wide characterization of m^6^A methylation patterns during TGEV infection. Our findings reveal distinct m^6^A methylation patterns between infected and control cells, and further analyses suggest that m^6^A-dependent modulation of transcript levels may critically contribute to orchestrating cellular responses during TGEV infection.

Here, our studies analyzed the m^6^A landscape in TGEV-infected and uninfected IPEC-J2 cells and identified 14,813 m^6^A peaks corresponding to 7728 genes. Most m^6^A-modified transcripts contained one or two peaks. Subsequent analyses revealed that these methylation sites were predominantly enriched in the CDS and 3′-UTRs, consistent with observations reported in other livestock and poultry species [40,41,42,43], indicating a high degree of evolutionary conservation in m^6^A distribution patterns. Notably, 42.81% and 44.06% of m^6^A sites in TGEV-infected and control cells, respectively, localized to CDS regions, supporting the notion that m^6^A may influence mRNA alternative splicing and translational efficiency [44]. Our findings align with previous reports [45] demonstrating that m^6^A peaks are more commonly enriched within 3′-UTRs than in other transcript regions. Given that the 3′-UTR plays essential roles in regulating mRNA stability, localization, translational efficiency, and protein-RNA interactions [46], the high prevalence of m^6^A deposition in this region suggests an important regulatory function during TGEV infection. In both control and TGEV-infected cells, m^6^A peaks were enriched in conserved GAACU or GAACC motifs, which serve as canonical recognition sites for methyltransferases. Although GGACU is widely recognized as a key m^6^A consensus sequence, only a subset of these motifs is methylated in vivo [47]. Moreover, the identification of novel methyltransferases and additional m^6^A-binding sequence preferences suggests that the classical RRACH motif alone cannot fully explain the distribution of m^6^A marks [48]. Together, these observations indicate that specific sequence motifs may be selectively recognized by m^6^A writers, erasers, or readers and likely contribute to the regulatory responses of IPEC-J2 cells during TGEV infection.

RNA m^6^A modification has emerged as an important post-transcriptional regulatory mechanism involved in cell differentiation, development, and host responses to pathogen infection. In the present study, 832 differential m^6^A peaks were identified between TGEV-infected and control groups, including 563 hypermethylated and 269 hypomethylated sites. Transcripts harboring hypomethylated m^6^A peaks were enriched in pathways such as Wnt signaling and axon guidance. KEGG analysis further revealed significant enrichment of both differentially methylated genes (DMGs) and differentially expressed genes (DEGs) within the Wnt signaling pathway. The Wnt pathway is a highly conserved regulatory cascade known to participate in the replication and pathogenesis of diverse viruses [49,50,51]. Previous work has shown that TGEV activates the Wnt/β-catenin signaling axis to enhance intestinal stem cell self-renewal and promote epithelial regeneration following infection [52]. Additionally, Wnt signaling has been implicated in tissue development and the suppression of intestinal inflammation through noncanonical signaling routes [53]. These findings, together with our methylation data, suggest that m^6^A modification may modulate TGEV-induced inflammatory responses by influencing the transcriptional output of Wnt-related genes. A positive association between m^6^A deposition and mRNA abundance was also evident in our dataset. Through integrative analysis of m^6^A-RIP-seq and RNA-seq, we identified 105 TGEV-related candidate genes marked by m^6^A modification. Notably, SOS2 emerged as a central gene in the PPI network and was linked to the mTOR signaling pathway, as highlighted through KEGG enrichment. SOS2 is a key upstream regulator of mTOR, which plays a crucial role in controlling autophagy, protein synthesis, and antiviral responses during multiple viral infections [54,55,56]. Previous studies demonstrated that leucine stimulates the expression of STAT1 and ISGs by activating mTOR signaling in IPEC-J2 cells, thereby offering protection against TGEV [57]. Taken together, these findings suggest that m^6^A modification may influence TGEV replication by modulating the SOS2-mTOR signaling axis. Although the mechanistic basis of m^6^A-dependent transcriptional regulation in pigs remains fragmentary, our findings represent promising candidates for understanding virus-induced metabolic dysregulation and may serve as potential biomarkers for disease. In future studies, we will not only use RNA interference to further validate the function of the *SOS2* gene at the cellular level, but also comprehensively investigate and verify the function and underlying mechanisms of *SOS2* in intestinal organoids by CRISPR-Cas9.

## 5. Conclusions

In this study, we characterized the m^6^A methylation landscape of TGEV-infected and control IPEC-J2 cells and demonstrated that m^6^A modification serves as a critical modulator of gene expression dynamics, particularly within the SOS2-mediated mTOR signaling pathway. These findings establish a foundation for further investigations into the epitranscriptomic mechanisms underlying TGEV infection and provide new insights into RNA-based regulatory processes in porcine viral diseases.

## Data Availability

The raw high-throughput m^6^A and RNA-seq data have been uploaded to the NCBI SRA repository under the BioProject ID: PRJCA051364.

## Supplementary Materials

The following supporting information can be downloaded at: https://www.mdpi.com/article/10.3390/vetsci13010010/s1. Table S1: Primer information for the RT-qPCR; Table S2: Reads statistics; Table S3: Diffpeak_statistic of m^6^A modification sites in genes in TGEV-treated IPEC-J2 cells compared with control cells (J2_TGEV vs. J2_MOCK); Table S4: Differentially expressed genes between J2_TGEV and J2_MOCK groups (J2_TGEV vs. J2_MOCK); Table S5: List of 105 genes that exhibited a significant change both at the m^6^A level and in mRNA transcript abundance in TGEV-treated IPEC-J2 cells as compared with control cells. Table S6: List of KEGG pathways obtained by enrichment analysis.

## Abbreviations

TGEV: transmissible gastroenteritis virus; m^6^A: N^6^-methyladenosine; m^5^C: 5-methylcytosine; m^7^G: N^7^-methylguanine; m^1^A: N^1^-methyl adenosine; stopC: stop codon; TSS: transcriptional start site; CDS: coding sequence; 3′-UTR: 3′-untranslated region; IPEC-J2: porcine intestinal epithelial cells; MeRIP-seq: methylated RNA immunoprecipitation sequencing; qRT-PCR: quantitative real-time PCR; TCID_50_: 50% tissue culture infective dose; CPE: cytopathic effects; DEGs: differential expression genes; DMGs: differentially methylated genes; GO: Gene Ontology; KEGG: Kyoto Encyclopedia of Genes and Genomes; SD: standard deviation; PPI: protein–protein interaction.
